# Intestinal carriage of vancomycin‐resistant *Enterococcus* spp. among high‐risk patients in university hospitals in Serbia: first surveillance report

**DOI:** 10.1186/s12941-021-00423-0

**Published:** 2021-03-20

**Authors:** Ana Janjusevic, Ljiljana Markovic Denic, Rajna Minic, Anita Grgurevic, Ivana Cirkovic

**Affiliations:** 1 Department of Bacteriology, Institute of Virology, Vaccines and Sera “Torlak”, Vojvode Stepe 458, 11152 Belgrade, Serbia; 2 Department of Epidemiology, Institute of Epidemiology, Faculty of Medicine, University of Belgrade, Visegradska 26a, PO Box 20, 11129 Belgrade, Serbia; 3 Department of Bacteriology, Institute of Microbiology and Immunology, Faculty of Medicine, University of Belgrade, Dr Subotića 1, 11000 Belgrade, Serbia

**Keywords:** VRE carriage, MLVA sreening, Antibiotic susceptibility, Serbia

## Abstract

**Background:**

The screening for intestinal carriage of vancomycin-resistant *Enterococcus* spp. (VRE) among high risk patients in the Balkan region and molecular epidemiology of VRE is insufficiently investigated, yet it could be of key importance in infection control. The aim of this study was to provide baseline data on VRE intestinal carriage among high-risk patients in Serbian university hospitals, to determine the phenotypic/genotypic profiles of the isolated VRE, to obtain knowledge of local resistance patterns and bridge the gaps in current VRE surveillance.

**Methods:**

The VRE reservoir was investigated using stool samples from 268 inpatients. Characterization of isolated VRE stains consisted of BD Phoenix system, genotypic identification, glycopeptide and quinupristin–dalfopristin (Q–D) resistance probing, virulence gene (*esp*, *hyl*, *efaA*, *asa1*, *gelE*, *cpd*) detection and MLVA. Biofilm formation was evaluated by the microtiter plate method.

**Results:**

VRE carriage prevalence among at-risk patients was 28.7%. All VRE strains were *van*A positive multidrug-resistant *Enterococcus faecium* (VR*fm*), harboring *erm*B-1 (38.9%), *esp* (84%), *efa*A (71.2%), *hyl* (54.5%), *asa*1 (23.4%), *gel*E and *cpd* (11.6%) each. Ability of biofilm production was detected in 20.8%. Genetic relatedness of the isolates revealed 13 clusters, heterogeneous picture and 25 unique MTs profiles.

**Conclusion:**

The obtained prevalence of VRE intestinal carriage among high-risk inpatients in Serbia is higher than the European average, with high percentage of multidrug resistance. The emergence of resistance to Q–D is of particular concern. Close monitoring of pattern of resistance and strict adherence to specific guidelines are urgently needed in Serbia.

## Introduction

Vancomycin resistant enterococci (VRE) have emerged as one of the most important health-care associated (HA) multidrug-resistant (MDR) pathogens, causing invasive infections, especially in severely ill and immunocompromised patients [1–3]. Limited therapeutic options for the treatment of VRE infections, the ability of VRE to survive in hospital environment, the capability to colonize the digestive tract of patients and the capacity for acquiring and transferring resistance genes along with the danger of vancomycin-resistant *Staphylococcus aureus* occurrence are the reasons that VRE was identified as an ESKAPE pathogen (*E*nterococcus faecium, *Staphylococcus aureus*, *Klebsiella pneumoniae*, *Acinetobacter baumanni*, *Pseudomonas aeruginosa* i *Enterobacter species*) [4] and recently selected as a high priority pathogen in the global priority pathogens list of the The World Health Organization for the development of new antibiotics [1–3, 5–7].

VRE colonization often precedes infection [8, 9] and numerous studies have identified hospital wards as places where VRE colonization poses the greatest risk to human health, in particular hematology, oncology, dialysis, intensive care units (ICUs), geriatrics and acute infectious diseasese wards [2, 3, 10–13]. Previous studies have shown that the prevalence of VRE colonization among hospital patients in Europe varies from 2 to 37% [3, 14].

Strategies to control the transmission of VRE in hospital settings include screening, early identification of colonization and the isolation of high risk patients [1, 2, 5, 9, 15]. The information about intestinal carriage and molecular epidemiology of VRE among at-risk patients in the Balkan region is scarce. Therefore, case-based surveillance [16] through which the baseline data can be obtained on the prevalence of VRE intestinal carriage as well as molecular epidemiology studies of circulating VRE stains in high-risk units are needed.

The first case of VRE in Serbia was reported in 2002 in the Clinical Center of Serbia (CCS), the largest tertiary-care teaching hospital in Serbia, which is also a major referal center for neighboring countries of ex-Yugoslavia (Montenegro, Bosna and Herzegovina) [17]. Sixteen years later, according to the data of the Central Asian and European Surveillance of Antimicrobial Resistance network [18], among invasive enterococci in Serbia vancomycin resistance was found in 54% of *Enterococcus faecium* and in 5% of *Enterococcus faecalis* isolates*.* These results were suggested to reflect hospital acquired infections and clone dissemination [18].

As hospital acquired VRE infections could be the tip of the iceberg [9] the aim of this study was to provide baseline data on VRE intestinal carriage among patients at high risk departments for VRE colonization in major Serbian university hospitals and to determine the full antibiotic susceptibility profiles along with frequency of resistance to individual drugs, biofilm production capacity and genetic relatedness of the isolated VRE. We also sought to bridge the gaps in current VRE surveillance in the Balkan region regarding screening data for pateints in high risk areas as part of a new concept of case-based surveillance [16] and to enhance the role of microbiology laboratory as a key partner in survaillance of antimicrobial resistance (AMR).

## Materials and method

### Study design and setting

The intestinal VRE reservoir was investigated by multicenter cross-sectional study in six hospital departments of university hospitals in Serbia over the period of 1.5 years (from June 2015 to January 2017): geriatrics, 29 beds; ICUs, 22 beds; hemato-oncology, 25 beds; acute infective disease, 21 beds; hemodialysis, 31 beds.

The sample size was calculated using Epi info™ 7 (CDC, USA) statistical software. Parameters used to calculate the sample size were: expected frequency of colonized patients with VRE strains in the hospital population of 18% [14], with a 95% confidence interval and acceptable margin of error of 5%. Accordingly, the sample size was calculated to be 227.

Participation was voluntary and comprised inpatients aged 18 years and older, of both sexes, who signed informed consent form prior to their inclusion in the study. The study was approved by The Ethical Boards of the included University hospitals: CCS, Zvezdara University Medical Centre (ZvUMC), Zemun University Medical Centre (ZmUMC). Important to note is that Serbia is a middle income Southeastern European country, with about seven million inhabitants, of which roughly 1/4 lives in Belgrade.

### Isolation

Stool samples for VRE testing were collected from 268 inpatients in sterile containers and were processed within 2 h after collection. Chromogenic agar medium (CHROMID®VRE, bioMerieux, France) was used for VRE screening. Stool samples were directly plated. In accordance with the manufacturers’ recommendations, plates were incubated at 36 ± 1 °C in ambient air, examined for growth after 24 h and 48 h and violet and blue-green colonies were presumptively identified as vancomycin resistant *Enterococcus faecium* (VRE*fm)* or vancomycin resistant *Enterococcus faecalis* (VRE*fs*), respectively. Three to four VRE colonies were inoculated into 1 mL of sterile Tryptic soy broth (TSB, Torlak, Serbia) and overnight cultures were microscopically examined for purity. Overnight cultures were placed in 10% glycerol and stored at − 72 ± 1 °C until future processing. Prior to testing, isolates were subcultured onto Columbia agar with 5% sheep blood (Torlak, Serbia).

### Identification to the species level and antimicrobial susceptibility testing

Identification and antimicrobial susceptibility testing were performed using the BD Phoenix™ automated microbiology system (BD, USA) with Gram Positive Combo Panels (PMIC/ID-94, BD, USA). Interpretation of results and quality control were performed in accordance with the latest version of the European Committee on Antimicrobial Susceptibility Testing (EUCAST) recommendations (v 10.0) [19].

### Molecular identification and detection of resistance and virulence genes

DNA isolation was performed from the pellet of 5 mL overnight cultures. The pellet was resuspended in 200 µL of TE buffer with 0.1 mm glass beads. After vortexing egg white lysozyme and achromopeptidase were added and incubated for 2 h at 36 ± 1 °C. Two freeze–thaw cycles were performed. Subsequently, 30 μl lysis buffer was added and diluted with 180 μl buffer (10 mM Tris–HCl, pH 8.5). Cell lysates were centrifuged at 16,000×*g* for 5 min. DNA from the supernatant was precipitated using ethanol and dissolved in water.

Multiplex polymerase chain reaction (PCR) was performed to detect genes for species identification (*ddl*_*E*_*. *_*faecium*_*, ddl*_*E*_*. *_*faecalis*_) and for detection of resistance to vancomycin (*van*A, *van*B, *van*C1, *van* C2/C3) [20–22]. Another multiplex PCR was performend to detect Quinupristin–dalfopristin (Q–D) resistance genes (*vat*D, *vat*E, *vgb*A, *erm*B-1) [23].

The presence of six virulence genes was tested in this study: *esp, hyl, efaA, asa1, gelE,* and *cpd* [24, 25]*.* In this analysis two multiplex PCRs were performed.

For PCR reactions Fast Gene Taq Ready Mix with dye (NIPPON Genetics, EUROPE GmbH) or Fusion Hot Start II High Fidelity Master Mix (Thermo Scientific, USA) were used as apropriate. Amplified products were analyzed with 1.5% w/v agarose gel. As molecular weight size marker 100 bp DNA Ladder (H3 RTU, NIPPON Genetics, EUROPE GmbH) was used.

### MLVA typing

VRE*fm* isolates were genotyped using Multiple-locus variable-number tandem-repeat (VNTR) analysis (MLVA) which was performed using a previously described method [26] with minor modification. An initial denaturation at 95 °C for 5 min was used for all cycles. VNTR-2 Touch Down (TD) PCR was done with decreasing annealing temperature for 0.7 °C at each cycle during the next 9 cycles and during the next 35 cycles, an annealing temperature of 63 °C was used. VNTR-9 TD PCR was done as follows: 10 cycles of 30 s at 94 °C, 30 s at 70 °C and 1 min at 72 °C with annealing temperature decreasing for 0.6 °C at each cycle during the next 9 cycles and during the next 35 cycles an annealing temperature of 64 °C was used. For VNTR-7, VNTR-8 and VNTR-10 PCR involving 35 cycles and an annealing temperature of 55 °C was used.

Cluster analysis of MLVA data was performed by BioNumerics software (version 7; Applied Maths) using UPGMA (Unweighted-pair group) clustering method with categorical coefficient of similarity. Cut-off value for clustering was set on 85% of similarity with a minimum of 2 members in a cluster and Simpson’s index of diversity was calculated. MLVA types (MTs) were assigned by comparison with the existing MLVA base [26]. For new combinations of MLVA profiles, new MTs were assigned. They were named according to the country of isolation (SRB) followed by incrising number obtained from phylogenetic analyses.

### Biofilm formation assay

The ability of isolated VRE to form biofilm was tested by microtiter plate method [27] in 96-well flat-bottomed polystyrene plates using TSB supplemented with 1% glucose and crystal violet staining. The absorbance of each plate was measured at 580 nm using a microtiter plate reader and the results were calculated according to Stepanović et al. [27].

## Results

A total of 268 inpatients were tested, of which 77 (28.7%) were intestinal carriers of VRE*fm* strains, with *vanA* genotypic profile and VanA phenotypic profile. The frequencies of VRE*fm* carriage among patients hospitalized in the aforementioned departments are shown in Table 1.

**Table 1:** Distribution of isolated Vancomycin-resistant *Enterococcus faecium* (VRE*fm*) stratified by investigated hospital departments

Department	VRE*fm*	
	n/N	%
Geriatrics	23/54	42.6
ICUs	16/40	40.0
Hemato-Oncology	22/79	27.9
Acute infectious diseases	10/44	22.7
Hemodialysis	6/51	11.7
Total	77/268	28.7

*ICUs* intensive care units, *n* number of VRE positive patients, *N* number of participants

The analysis of antibiotic susceptibility, shown in Table 2, exposed high prevalence of multidrug-resistant VRE strains. In addition to vancomycin and teicoplanin resistance, 23.4% (18/77) VRE*fm* isolates were resistant to all remaining antimicrobial drugs, except linezolid and tigecyclin to which all the isolates were susceptible. Additionaly, 38.9% of VRE*fm* strains were resistant to Q–D.

**Table 2:** Antibiotic resistant profile of Vancomycin-resistant *Enterococcus faecium* (VRE*fm*) isolates (N = 77)

Antimicrobial drug	VRE*fm* isolates	
	Resistant	
	n	%
AMP	71	92.2
IMP	71	92.2
CIP	75	97.4
LEV	75	97.4
GEN-HLS	69	89.6
STR-HLS	73	94.8
TEI	77	100
Q–D	30	38.9
TIG	0	0
LIN	0	0

*n* number of VRE*fm* isolates resistant to tested antimicrobial drug, *AMP* ampicillin, *IMP* imipenem, *CIP* ciprofloxacin, *LEV* levofloxacin, *GEN-HLS* gentamicin-high level aminoglycoside resistance, *STR-HLS* streperomicin-high level aminoglycoside resistance, *TEI* teicoplanin, *Q–D* quinupristin–dalfopristin, *TIG* tigecyclin, *LIN* linezolid

Phenotypic resistance profiles of the isolated VRE*fm* strains are shown in Table 3.

**Table 3:** Phenotipic profile of isolated Vancomycin-resistant *Enterococcus faecium* (VRE*fm*)—antimicrobial susceptibility pattern

Antimicrobial susceptibility pattern	Resistant	
	n	%
AMP IMP CIP-LEVO GEN-HLS STR-HLS TEI	48	62.3
AMP IMP CIP-LEVO GEN-HLS STR-HLS TEI Q–D	18	23.4
CIP-LEVO GEN-HLS STR-HLS TEI Q–D	4	5.2
AMP IMP CIP-LEVO STR-HLS TEI Q–D	2	2.6
AMP IMP CIP-LEVO GEN-HLS TEI Q–D	2	2.6
CIP-LEVO GEN-HLS TEI	1	1.3
AMP IMP TEI Q–D	1	1.3
TEI Q–D	1	1.3
Total	77	100

*AMP* ampicillin, *IMP* imipenem, *CIP* ciprofloxacin, *LEV* levofloxacin, *GEN-HLS* gentamicin-high level aminoglycoside resistance, *STR-HLS* streperomicin-high level aminoglycoside resistance, *TEI* teicoplanin, *Q–D* Quinupristin–dalfopristin

Biofilm quantitative test showed that 20.7% (16/77) bacterial strains were biofilm producers with different capacity. Biofilm production capacities of given pathogens are shown in Table 4.

**Table 4:** Phenotipic profile of isolated vancomycin-resistant *Enterococcus faecium* (VRE*fm*)—biofilm production capacity

Biofilm production capacity	N		%
Yes	16	20.8	
Weak	9	11.7	
Moderate	4	5.2	
Strong	3	3.9	
No	61	79.2	
Total	77	100	

weak biofilm producer—category 1, +; moderate biofilm producer—category 2, ++; strong biofilm producer—category 3, +++; no—no biofilm formation

Molecular genetic analysis for molecular confirmation of Q–D resistance was performed on DNA samples obtained from purified isolates. The analysis confirmed that all isolates showing phenotypic resistance carried the *erm*B-1 gene. Other genes associated with resistance to Q–D (*vat*D, *vat*E, *vgb*A) were not detected. Genetic analysis of the presence of virulence genes revealed that 84% VRE*fm* isolates harbored the *esp* gene, while three isolates harbored all tested virulence genes (Table 5).

**Table 5:** Characteristics of isolated Vancomycin-resistant *Enterococcus faecium* (VRE*fm*) – genotypic profile

Genotypic profile	N	%
Identification genes		
*ddl*_*E. faecium*_	77	100
*ddl*_*E. faecalis*_	0	0
Vancomycin resistance genes		
*vanA*	77	100
*vanB*	0	0
*vanC(C1and C2/3)*	0	0
Q–D resistance genes		
*vat*D	0	0
*vat*E	0	0
*vgb*A	0	0
*ermB*-1	30	38.9
Virulence genes		
*esp*	65	84
*efaA*	55	71.2
*hyl*	42	54.5
*gelE*	9	11.6
*asa1*	18	23.4
*cpd*	9	11.6
Number of virulance genes		
0	8	10.4
1	4	5.2
2	20	26.0
3	34	44.0
4	4	5.2
5	4	5.2
6	3	4.0

*ddl*_*E. faecium*_, d-alanine–d-alanine ligase gene specific for *E. faecium*;* ddl*_*E. faecalis*_, d-alanine–d-alanine ligase gene specific for *E. faecalis*;* van A*, type A vancomycin resistance; *vanB*, type B vancomycin resistance; *vanC (C1and C2/3)—*type C vancomycin resistance; *vat*(D) and *vat*(E) , streptogramin A resistance; *vgb*(A) and *erm*B-1*-* streptogramin B resistance; *esp*, Enterococcal surface protein;* hyl*, hyaluronidase;* efaA*, cell wall adhesine;* asa1*, aggregation substance; *gelE*, gelatinase; *cpd*, sex pheromones

In order to assess the genetic relatedness of the VRE*fm* isolates a standard MLVA analysis was performed on isolated DNA samples. A total of 72/77 (93.5%) isolates generated a MLVA profile, while MLVA profiles could not be assigned for 5/77 (6.5%) isolates. MLVA revealed 29 different MTs, of which 25 were not previously detected and had unique profiles. Simpson´s index of diversity was 94%.

**Fig. 1:**
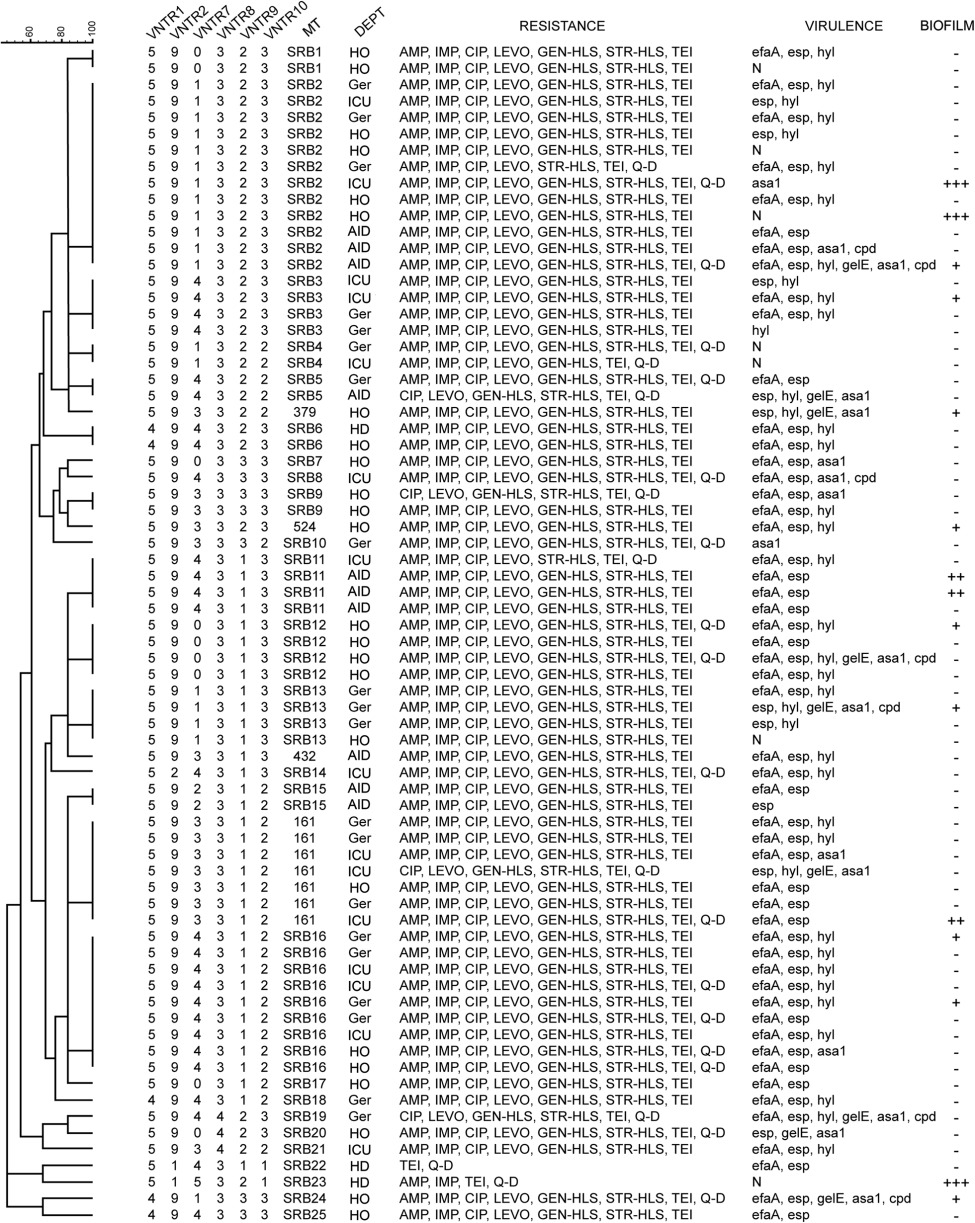
Dendogram of Vancomycin-resistant *Enterococcus faecium* isolates using Unweighted pair group clustering method. *VNTR*, variable-number tandem-repeat; MT-MLVA type; HO, Haematology-Oncology; Ger, Geriatrics; ICU, Intensive Care Units; AID, Acute Infection Diseases; HD, Haemodyalisis; AMP, Ampicillin; IMP, Imipenem; CIP, Ciprofloxacin; LEV, Levofloxacin; GEN-HLS, Gentamicin-high level aminoglycoside resistance; STR-HLS, Streperomicin-high level aminoglycoside resistance; VAN, Vancomycin; TEI, Teicoplanin; Q–D, Quinupristin–dalfopristin; *ddl*_*E. faecium*_, d-alanine–d-alanine ligase gene specific for *E. faecium*;* ddl*_*E. faecalis*_, d-alanine–d-alanine ligase gene specific for *E. faecalis*;* van A*, type A vancomycin resistance; *vanB*, type B vancomycin resistance; *vanC (C1and C2/3)—*type C vancomycin resistance; *vat*(D) and *vat*(E), streptogramin A resistance; *vgb*(A) and *erm*B-1, streptogramin B resistance; *esp*, Enterococcal surface protein*; hyl*, hyaluronidase*; efaA*, cell wall adhesine;* asa1*, aggregation substance; *gelE*, gelatinase; *cpd*, sex pheromones; N, no virulence genes; +, weak biofilm producer; ++, moderate biofilm producer; +++, strong biofilm producer;–, no biofilm production

Genetic relatedness analysis of the isolates revealed 13 clusters which comprised 56/72 (77.7%) of the isolated VRE*fm* strains (Fig. 1). Three of 13 clusters included 12 (SRB2), 9 (SRB16) and 7 (MT161) isolates each, while the 10 remaining clusters included 2 to 4 isolates. The remaining 16/72 (22.2%) VRE*fm* isolates had unique genotypes and were not clonally related to the other isolates. Next we sought to determine the association of genotype with the location of inpatient from which the VRE*fm* was isolated. We detected that the isolates belonging to the larger clusters were dispersed among different hospital departments (Fig. 1). Interestingly, isolates belonging to the 3 minor clusters (SRB6, SRB9, SRB12) originated from hematology department only.

## Discussion

In the last 15 years, VREs derived as a major cause of HA infections worldwilde due to intrinsic and acquired resistance to various classes of antimicrobial drugs, scarcity in treatment options and high tendency to become endemic in the hospital environment [28]. European Centre for Disease Prevention and Control points out an increasing trend in the number of VRE*fm* infections and VRE*fm* associated deaths in European countries and its contribution to European health burden of antimicrobial resistance, highlighting the urgency for better understanding molecular epidemiology of circulating VRE [28]. VRE colonization often precedes infection and selective screening of at-risk patients for VRE colonization is one of the infection controling measures recommended for prompt identification and isolation of carriers, thus being the crutial step in control of patient-to-patients transmission [2, 3, 8, 9].

This study presents the first case-based surveillance report on the prevalence of VRE intestinal carriage among high-risk inpatients in Serbia and partially in the ex-Yugoslav region. The intention was to broaden awareness and increase knowledge on VRE intestinal carriage in the Balkan region as well as to provide baseline data regarding molecular epidemiology and genetic diversity of circulating VRE strains in Serbia.

VRE carriage was found in 28.7% of the study population. This frequency is high compared to low prevalence European countries, e.g. the Netherlands (2%) and Belgium (3.5%) but similar to frequencies reported in studies from Ireland (19.1%), United States of America (USA) (33%) and certain regions of France (37%) [3, 14, 29]. However, comparison of the data form different countries is very difficult and should be done carefully as there are many variables to be taken into account. For example, there could be differences in the study period, the primary diagnosis, in the particular hospital units investigated, in the type of the analyzed specimens and in the media used for isolation. Additionally there could be differences in reporting of acquired and intrinsic resistance profiles which may have significant impact on the obtained results.

In this study, we used the direct plating method of stool specimens onto selective chromogenic agar medium for the detection of the acquired VRE*fs* and VRE*fm* strains. Several studies [3, 30] evaluated usefulness of the selective chromogenic media in the detection of VRE colonization, which can explain the high rate of recovery of VRE in our study.

All detected VRE strains were VRE*fm* with *vanA* genotypic profile and VanA phenotypic profile, which is typical for European counties [1, 2]. Similar to the study of Whelton et al. [29], neither VRE*fs* nor *Enterococcus gallinarum/casseliflavus* were isolated in our research. Predominance of multidrug-resistant VRE*fm* may indicate that the strategy of making hospital environment its novel ecological niche has been successfully mastered by the bacteria. This is in line with previous conclusions that multidrug resistant *E. faecium* is a dominant reservoir of acquired vancomycin resistance in hospitals, particularly among immunocompromised patients, resulting in dissemination of resistance genes among the bacterial population upon selective antibiotic pressure [2, 5].

Interestingly, high rate of resistance to Q–D (38.9%) in VRE*fm* isolates was found. Q–D is a streptogramin, antimicrobial drug licensed for treatment of infections caused by MDR bacteria, including VRE*fm*. Although resistance to this drug among VRE*fm* is known, it is rare in human isolates [31]. Moreover, Q–D is not licensed for clinical use is Serbia. Thus, the high frequency of resistance to Q–D among VRE*fm* strains in Serbia is of special concern for two reasons. Firstly, the ability of VRE*fm* strains resistant to Q–D to spread and cause healthcare-associated outbreaks is potentially high. Secondly, treatment options for infections caused by these strains are narrowed. We found that all VRE*fm* isolates resistant to Q–D harbored the *erm*B-1 gene, that conferred resistance to macrolides, lincozamine and streptogramine B, which is in line with previous findings [31]. Considering that Q–D is not licensed for clinical use in Serbia and that it has never been a part of therapeutic protocols in Serbia, possible explanation for Q–D resistance in addition to vancomycin resistance is the exchange of resistance genes between *E. faecium* from human and animal sources, as resistance to Q–D is common in isolates retrieved from animals and is linked to the use of virginamycin as growth promotion factor [23, 31].

In order to better understand VRE colonization and its implication in infection, we studied virulence factors. Obtained results showed that almost all tested virulence genes (*esp*, *hyl*, *efa*A, *asa*1, *gel*E, *cpd*) were present in analysed VRE*fm* strains. Additionally, 58.4% of the isolates were found to carry a minimum of 3 known virulance genes, which is in contrast to the findings of Billström [32] where only 2% of isolates carried multiple virulence genes. Our data showed that VRE*fm* with all the virulence factors seems to be equipped to survive and spread in hospital environment. This ability to collect various virulence genes may be explained by adaptive mechanisms, e.g. “genetic capitalism” due to the gene transfer and recombination [33–35]. Recently, Freitas et al. [36] has shown that the number of virulence genes present in VRE*fm* correlate well with ampicillin-resistant phenotype and that VRE*fm* strains with higher ampicillin minimum inhibitory concentration values have higher number of virulence genes, which contributes to their pathogenicity. Almost all VRE*fm* strains from our study were resistant to ampicillin and most of them contained several virulence genes and therefore might pose a serious risk for infection in at-risk patients.

Finaly, in our study we performed MLVA typing, fast, cheap, easy-to-use, PCR-based method recommended as an initial rapid screening typing tool for the analysis of phylogenetic relatedness of isolated VRE*fm* in hospital settings [26, 37]*.* The largest isolated clusters (SRB2, MT 161 and SRB16) represent single-locus variant (SLV) or double locus variants of MT- 340 and MT-159, known to cause infections in hospitalizied patients in Serbia [38, 39]. Namely, MLVA-C1 genogroup comprises the majority of epidemic and clinical VRE*fm* isolates, with MT-1 and its SLV MT-159, being the most common types associated with hospital outbreaks and invasive infections in the last decade in Europe [39]. Therefore, our results might indicate an evolution in hospital-adopted clones which might happen sporadically. Simpson’s index of diversity demonstrated high diversity among the isolates, implying phylogenetic unrelatedness. Being that we found five isolates which could not be classified by MLVA, possibly as a result of lacking one to three VNTR loci, as well as 25 unique MTs, one could speculate that the origin of vancomycin resistance in *E. faecium* in our study is due to the horizontal gene tranfer and selective antibiotic pressure [33, 34, 40]. Indeed, high prevalence of VRE*fm* was found in geriatrics departments where vancomycin is a common agent for *Clostridioides difficile* infection treatment. Also, broad-spectrum antimicrobial therapy is the most common therapeutic protocol in ICUs, while vancomycin is commonly used as part of empiric therapy of febrile neutropenia in hematological patients [10, 13]. Hence, unlike the rest of Europe, where the origin of VRE in hospitals is connected to the usage of avoparcin in farm animals, in Serbia we noticed a different scenario, likely similar to that in the USA, where VRE in hospitals is due to the overuse of vancomycin [2].

Although definitive proof of patient-to-patient transmission requires high resolution typing techniques like multilocus sequence typing and whole genome sequencing [9, 41], we consider MLVA to be a reliable typing method in the context of infection control surveillance where primary focus is the exclusion of clonal relatedness among isolates and timely alert if an outbreak investigation is about to commence.

## Conclusion

The obtained prevalence of VRE intestinal carriage among high-risk inpatients in Serbia is higher than the European average, with high percentage of multidrug-resistance and the ability of biofilm production. Various virulence genes might affect the pathogenicity of the strains. Reporting full antimicrobial resistance profiles along with the frequency of resistance to individual drugs for at-risk population is a change in approch to reporting AMR data and a step closer to the new concept of case-based surveillance of AMR. Of particular concern is the emergence of resistance to Q–D that has never been licensed for clinical use in Serbia nor has ever been a part of therapeutic protocols in our country. The illicit usage of antibiotics in animal farming could be implicated. MLVA revealed polyclonal setting with 25 unique MT profiles that were most likely selected through antibiotic pressure. This study also contributes to the investigation of VRE*fm* genotype distribution in the neighboring countries within the Balkan peninsula. Close monitoring of the pattern of resistance, implementation of specific guidelines, cleaning procedures and antibiotic stewardship policy, as well as the introduction of VRE screening among at-risk inpatients, as part of active surveillance are urgently needed in Serbia.

## Data Availability

All data generated or analyzed during this study are included in this published article.

## Abbreviations

AMR: Antimicrobial resistance
CCS: Clinical Center of Serbia
EUCAST: European Committee on Antimicrobial Susceptibility Testing
HA: Health-care associated
ICUs: Intensive care units
MLVA: Multiple-locus variable-number tandem-repeat analysis
MTs: MLVA types
PCR: Polymerase chain reaction
Q–D: Quinupristin–dalfopristin
SLV: Single-locus varian
SRB: Serbia
TE buffer: Tris EDTA buffer
TSB: Tryptic soy broth
TD PCR: Touch down PCR
UPGMA: Unweighted-pair group
USA: Unites States of America
VNTR: Variable-number tandem-repeats
VRE: Vancomycin resistant enterococci
VRE*fm*: Vancomycin resistant *Enterococcus faecium*
VRE*fs*: Vancomycin resistant *Enterococcus faecalis*
ZvUMC: Zvezdara University Medical Centre
ZmUMC: Zemun University Medical Centre
